# Synthetic sticky bone grafts enhance bone regeneration: a preclinical evaluation in rat models

**DOI:** 10.1590/1678-7757-2025-0108

**Published:** 2025-06-13

**Authors:** Lei LI, Haojie LIN, Siyu JIN, Shuchang HU, Wei SUN, Wei JI

**Affiliations:** 1 Wuhan University School & Hospital of Stomatology State Key Laboratory of Oral & Maxillofacial Reconstruction and Regeneration Wuhan China Wuhan University, School & Hospital of Stomatology, State Key Laboratory of Oral & Maxillofacial Reconstruction and Regeneration, Key Laboratory of Oral Biomedicine Ministry of Education, Hubei Key Laboratory of Stomatology, Wuhan, China.; 2 Wuhan University School & Hospital of Stomatology Department of Implantology Wuhan China Wuhan University, School & Hospital of Stomatology, Department of Implantology, Wuhan, China.

**Keywords:** Osteogenesis, Bone regeneration, Bone substitutes

## Abstract

**Objectives:**

Deproteinized bovine bone minerals (DBBMs) are effective for bone regeneration. However, their limited plasticity can hinder extensive bone defects treatment. This study aimed to develop a composite bone grafting material that is easy to deploy surgically and promotes robust bone regeneration.

**Methodology:**

DBBM particles were mixed with a clinical-grade gelatin-based hemostatic gel (w/v ratio of 2/3) to create a composite material referred to as synthetic sticky bone (SSB). Structural properties were assessed using confocal laser scanning microscopy and scanning electron microscopy. To evaluate bone regenerative capacity, 20 male Sprague Dawley rats (eight to ten weeks old) with critical-size jawbone defects were treated with SSB, DBBM, or gelatin gel alone, with an empty defect as a control. Samples were collected at two and four weeks for microcomputed tomography (μCT) analysis of bone volume/total tissue volume (BV/TV), trabecular thickness (Tb. Th), trabecular number (Tb. N), and trabecular separation (Tb. Sp). Histological analyses were conducted to examine material remnants and bone formation.

**Results:**

SSB showed a binary paste-like composite property with enhanced injectability and plasticity. μCT and histological assessments confirmed that the SSB-treated group had significantly greater new bone formation compared to the DBBM-treated group after four weeks.

**Conclusions:**

SSB, which is a paste-like composite of DBBM particles, and a clinical-grade gelatin-based hemostatic gel demonstrated improved structural plasticity and enhanced bone regeneration, offering a promising solution for treating extensive irregular bone defects.

## Graphical abstract

**Figure:**
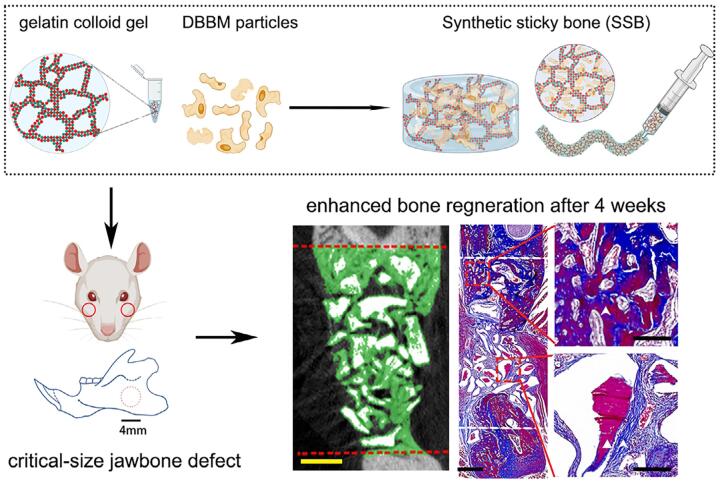

## Introduction

Guide bone regeneration (GBR) is a widely employed surgical technique that significantly enhances the success rates and longevity of dental implants. In this procedure, bone filler materials are crucial for facilitating new bone formation. From a biophysical perspective, bone fillers that mechanically mimic the natural extracellular matrix (ECM) and adapt to the irregular shapes and local mechanical stimuli at the defect site are essential for effective bone regeneration.^1^

Deproteinized bovine bone minerals (DBBM) are among the most common of the various bone filler materials used for GBR due to their well-documented osteoconductivity and porous structure, which resembles cancellous bone.^2-4^ However, limitations such as inadequate angiogenesis and delayed bone formation have raised concerns regarding the clinical application of DBBM.^1,5^ Additionally, current DBBM particles show insufficient plasticity, complicating their ability to maintain stable shapes and spaces, particularly in extensive bone defects.^6^ Therefore, the intraoperative handling properties of bone grafting materials are critical for their clinical applicability.

Recently, the “sticky bone” technique has emerged as an innovative approach to address such clinical challenges. This method combines DBBM with platelet-rich fibrin (PRF) to create a composite graft that leverages the osteoconductivity of DBBM while enhancing handling properties. The addition of PRF not only improves the cohesiveness and moldability of DBBM but also facilitates the delivery of growth factors that promote angiogenesis and tissue ingrowth.^7^ Despite its promising biological properties, the sensitivity of the technique and the limited availability of PRF raise concerns about its use in large defects.^8^ Furthermore, the lack of standardized protocols for PRF application also adversely affected treatment outcomes in various clinical settings.^9^ Therefore, from the clinical application perspective, it is necessary to find an operator-friendly and biocompatible method to improve the plasticity of DBBM.

Colloidal gels, which are composed solely of colloidal particles, represent a promising class of injectable biomaterials due to their viscoelastic properties and self-healing abilities.^10,11^ The dynamic and adaptive nature of colloidal gel networks fosters an excellent microenvironment for tissue ingrowth and cellular activity.^10^ Gelatin-based colloidal gels have shown promise in biomedical applications due to their sol-gel phase transition, moisturizing effects, biocompatibility, and structural similarity to the native ECM.^10,12,13^ Although the compressive strength of bare gelatin-based gels is inadequate for load-bearing applications, their viscoelastic properties make them suitable as lubricants for incorporating reinforcing agents.^10^

DBBM combined with gelatin-based colloidal gels have been extensively studied recently but most research remains at the preclinical evaluation stage, with few studies translated into clinical-grade products.^10^ We propose a “synthetic sticky bone” (SSB), which is an off-the shelf and easily prepared composite grafting material that combines clinical-grade gelatin-based colloidal gels with DBBM particles. We hypothesize that the SSB composite will provide enhanced handling properties, mechanical robustness and improved bone regenerative capacity. We performed microscopic assessments to visualize the interconnective structure of the composite grafting materials. Furthermore, we assessed the adaptability and bone regenerative capacity of the composite grafting materials *in vivo* using a critical-size jawbone defect model in rats, the lowest species which could provide it with similar biological characteristics that closely resemble human ones.^14^

## Methodology

### Preparation of composite grafts

**Figure 1:**
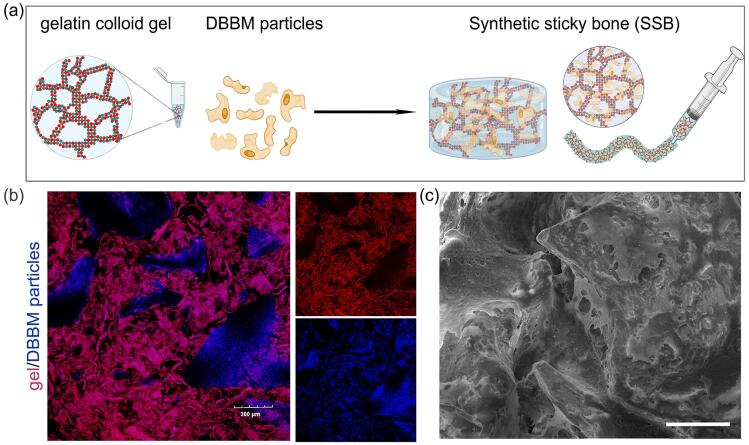
Preparation and characterization of the synthetic sticky bone (SSB). (a) Schematic illustration of the SSB preparation method, creating an injectable material made of hemostatic gelatin-based colloidal gel and deproteinized bovine bone mineral (DBBM) particles. Note: This figure was created with BioRender.com. (b) Confocal microscopic images of the SSB, indicating the gelatin-based colloidal gel (red) interacting with DBBM particles. Red = autofluorescence of gelatin at 580 nm channel; Blue = DAPI stained DBBM particles detected at 405nm, Scale bar = 200 μm. (c) Scanning electron microscopic images of the SSB composed of gelatin-based colloidal gel and DBBM particles. Scale bar = 200 μm.s

Clinical grade absorbable hemostatic gel (Colloidose^TM^, Huanova Biotech, Shenzhen, China), were mixed with saline at a mass fraction of 5% (w/v) to make gelatin-based colloid gel following the manufacturer’s instructions. Then, DBBM particles (Geistlich Bio-Oss^®^, Geistlich Pharma AG, Wolhusen, Switzerland) were mixed with the gelatin-based colloidal gel (w/v = 2/3) to form the paste-like synthetic sticky bone graft (SSB) (Figure 1A, Supplementary Video 1).

### Confocal laser scanning microscopy

To visualize the component distribution with SSB, the fluorescently tagged composite graft containing DAPI (Thermofisher) stained DBBM particles were immersed in phosphate buffered saline (PBS, pH 7.4) and imaged using an Olympus Fluoview 1200 confocal laser scanning microscope. The images were processed using ImageJ software (National Institutes of Health, USA), with adjustments for contrast, brightness, and color balance to obtain optimum visual representation of data.

### Scanning electron microscopy

SSB, DBBM particles and gelatin colloid gel were fixed in 3% glutaraldehyde for 20 minutes. The samples were rinsed three times with phosphate-buffered saline (PBS) and dehydrated via a series of increasing ethanol concentrations, ranging from 30% to 100%. Then, the samples underwent critical point drying using a Tousimis Samdri-795 apparatus to remove ethanol. The dehydrated samples were then mounted on carbon adhesive tape and coated with gold to create a conductive surface. All scanning electron microscopy (SEM) images were captured at an accelerating voltage of 20 kV using a VEGA 3 LMU instrument (Tescan, Czechoslovakia).

### *In vivo* evaluation of jawbone critical-size bone defect healing

#### Animal experiment design

Twenty male Sprague Dawley rats (eight to ten weeks old) with mean weight of 250g were purchased from the Centre for Disease Control of Hubei Province, China. All experiments with animals were conducted in compliance with the ARRIVE 2.0 guidelines and were approved by the Animal Ethics Committee of the School of Stomatology, Wuhan University (No. S07922090A). Prior to experiments, the animals were quarantined and caged for five to ten days for acclimatization. The experiments were conducted in an SPF-level laboratory with laminar airflow.

**Figure 2:**
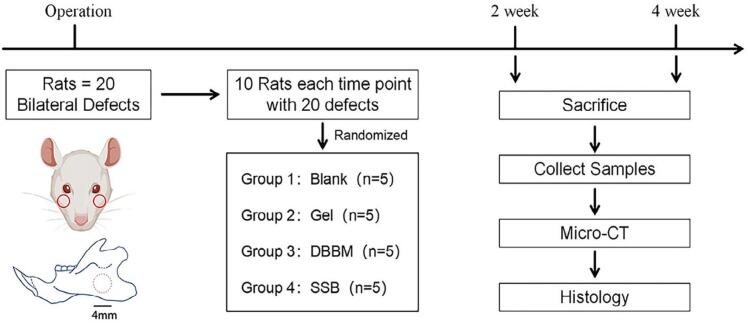
Schematic flowchart of the experiment. Note: This figure was created with BioRender.com.

We used a 4 mm-diameter full-thickness penetrating defect in the rat mandible ramus as the critical size defect model for investigating regeneration in the craniomaxillofacial region.^15^ Twenty rats were used to minimize the number of animals, and bilateral defects were created in one rat. The defects were randomized for the four groups of treatments following a randomization sequence created in Excel: ① SSB (10 mg DBBM particles mixed in 15 µL of colloidal gel); ② Gel (15 µL of colloidal gel); ③ DBBM (10 mg DBBM particles); and ④ empty defect (blank control). After two and four weeks, samples were harvested for radiographic and histological assessments (Figure 2).

#### Sample size calculation

To reduce the number of animals in the experiment, we calculated the sample size for each group according to a previous study that performed a similar assessment,^16^ which revealed that the implantation of DBBM alone could induce 21±2% of new bone formation in the defect area. We expected that with the addition of gelatin-based colloidal gel, the resulting SSB could reach 20% of the increase in new bone formation. Therefore, the minimal number of samples for each group was calculated as n=4 following the previous description,^17^ with the probability of Type-I error (alpha) set as 0.05, and power set as 80%. Considering the possible follow-up loss of the samples, the sample size was set as n=5 for each group at each time point. Hence, the minimal total number of animals used is calculated as: N=5 (sample size) * 4 (group number) * 2 (time points) / 2 (bilateral defects) = 20 .

#### Surgical procedure

The animals were anesthetized with isoflurane inhalation, supplemented by an intraperitoneal injection of a cocktail comprising zoletil (50 mg/kg) and xylazine (10 mg/kg). Once deeply sedated, isoflurane anesthesia was maintained continuously via facemask at 1.5-5% in oxygen. The surgical sites on both sides of the mandible were shaved and prepared in a sterile manner using 10% Betadine and 70% ethanol swabs.

A critical-size mandibular defect with 4 mm diameter was created according to established protocols.^18^ In brief, a 1-cm full-thickness skin incision was made 1 cm above the inferior border of the mandible to access the subcutaneous tissue followed by blunt dissection of the masseter and digastric muscle. Then, after stripping and periosteum of the mandibular ramus, a standard bi-cortical penetrating defect was made using a trephine drill (diameter = 4mm) and irrigated with 0.9% saline solution. The defects were then randomly assigned to one treatment group (n=5 for each group)*.* After filling the defect with the material, we carefully repositioned the periosteum over the defect and subsequently performed layered suturing of the muscles and skin using zero to four absorbable sutures.

#### Post-surgery care

After surgery, each rat received subcutaneous injections of 5 mg/kg ceftiofur and 2.5 mg/kg flunixin meglumine for five days to prevent infection and pain management. All animals were closely monitored daily for the following conditions: (1) any signs of pain or distress including squinting of eyes, decreased food and water intake, and abnormal breathing pattern; (2) signs of redness and swelling, exudates or other signs of infection at the incision site. A fluidic diet was provided for the first two days. After two and four weeks of surgery, the rats were euthanized via deep diethyl ether inhalation followed by cervical dislocation, in accordance with the institutional animal ethics guidelines. Then the mandibles were dissected. Each sample was fixed with 4% paraformaldehyde overnight at 4 and stored in 70% alcohol for further assessments.

## Quantitative analysis of microcomputed tomography (μCT) reconstructions

The harvested samples (n=5 for each group) were all scanned using SkyScan 1276 μCT system (Bruker). An operation voltage of 60 kV was used along with a 0.5 mm aluminum filter. Exposure time of 1000 ms with isotropic voxel size of 10 μm were used to acquire image. NRecon software (Bruker) was used to reconstruct the datasets regarding individual sample, and Data Viewer software (Bruker) was used to adjust angles to obtain a coronal vision for display and analysis. Next, as our previous report,^19,20^ CTAn (Bruker) software was used for 3D quantification, which employed a three level of automatic Otsu segmentation algorithm on the individual 2D slice in which *de novo* formed bone could be segmented from the background. The region of interest (ROI) was delineated from the first slice containing the defect and moved distally until the defect area disappeared. Within the ROI, bone volume/total tissue volume (BV/TV), trabecular thickness (Tb. Th), trabecular number (Tb. N), and trabecular separation (Tb. Sp) were calculated. The μCT assessments were performed by an independent researcher (S. Jin) who had no information about the sample.

## Histology and histomorphometry

All explants were decalcified in a 10% ethylene diamine tetra-acetic acid (EDTA)/PBS solution (pH 7.5) for 14 days, embedded in paraffin, and sectioned (7 μm in thickness) using a microtome (Leica). The sections were stained with Hematoxylin-Eosin (HE) (Beyotime, #G1005) and Masson Trichrome (Solarbio, #G1340) to assess general histology and the *de novo* formed bone, respectively. Sections were photographed using a Panoramic Digital Slide Scanner (3DHistech, Budapest, Hungary).

Histomorphometric analysis was performed on Masson Trichrome staining sections. Two sections per sample were measured, and five biological samples per group (n=5) were used in histomorphometric analysis. The analysis was performed by an experienced independent researcher (H. Lin) who had no information about the sample. In brief, the sections were scored using BoneJ plugin in Fuji, which recognize bone tissue from the fibrous tissue and scaffold materials based on RGB values from highly magnified digitalized images.^21^ Manual corrections were also applied to ensure the precise selection of newly formed bone tissue within the defect area.

The amount of bone formation was determined: bone formation area in the defect area (μm^2^) / cross-sectional distance (μm).^22^

## Statistical analysis

All numerical data were represented as mean ± standard deviation (SD), and data are plotted as individual data points with bars representing the mean value. Statistical analysis was performed using GraphPad Prism 8.0.2. (GraphPad Software Inc., San Diego, CA, USA). Shapiro-Wilk test was used for normal distribution. A non-paired unequal variance student’s *t*-test or Mann-Whitney test was used for two group experiments. Statistical significance is indicated on all tables and graphs as follows: * p<0.05. ** p <0.01, *** p<0.001, and n=5 if nothing else is indicated.

## Results

### The paste-like synthetic sticky bone graft (SSB) demonstrated adaptability and injectability

SSB, a paste-like material, showed morphological adaptability and injectability, which are attributed to the viscoelastic properties of the gelatin colloid gel. Such characteristics enable surgeons to eject the SSB from a syringe into the defect site, and fit into a specific shape (Figure 1a, and Supplementary Video 1).

**Figure 3:**
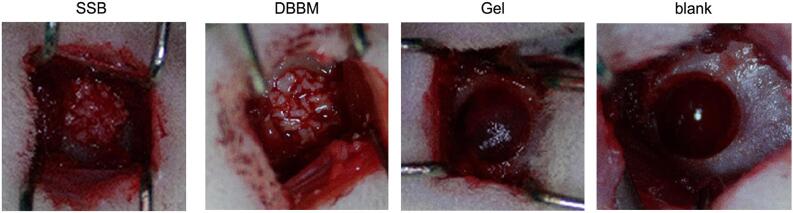
Rat critical-size jawbone defects treated with the SSB, DBBM particles, and gelatin-based colloidal gel (Gel), respectively. Empty defect (blank) was set as negative control.

Microscopic assessment revealed a strong adhesive interaction between the fibrous gel and DBBM particles (Figure 1b). Scanning electron microscopy further revealed that SSB consisted of a gelatin hydrogel network that penetrated the encapsulated DBBM particles (Figure 1c). When tested in well-established rat mandibular critical-size defect, SSB showed greater stability fitting within the defect compared to the DBBM particles and gelatin colloid gel alone, which were more susceptible to displacement from the defect area (Figure 3).

### SSB enhances bone regeneration in critical-size jawbone defect

**Figure 4:**
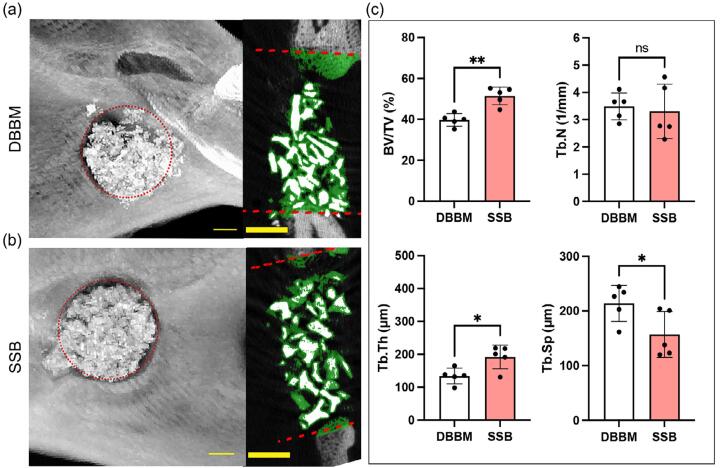
Microcomputed tomography (μCT) of Rat critical-size jawbone defects treated with the SSB, DBBM at postoperative 2 weeks. (a-b) Representative reconstructions from μCT are shown for the rat critical-size jawbone defect treated with DBBM (a) and SSB (b); green color indicates the newly formed bone in the defect area. Scale bar = 1mm; (c) Quantitative analysis of bone volume/tissue volume (BV/TV), trabecular thickness (Tb. Th), trabecular separation (Tb. Sp), and trabecular number (Tb. N) in defect sites treated with DBBM and SSB (n=5). Student’s t test was used for the statistical analysis. ns indicates no significance. *p<0.05. **p<0.01.

Mandibles were harvested at two and four weeks postoperative, after which microcomputed tomography reconstructions of the samples were performed. Representative μCT images revealed that, after two weeks, there was an empty defect in both blank and gelatin groups (Supplementary Figure 1). In contrast, SSB and DBBM particles were observed within the defect area, accompanied by the *de novo* bone formation surrounding the particles (Figure 4a-b). Quantitative analysis indicated that the BV/TV in the SSB group was significantly higher (51.45±4.31%) compared to the DBBM group (39.74±3.10%). Additionally, the SSB group showed a trabecular thickness of 192 ± 36 μm in the *de novo* bone, which was significantly greater (p=0.017) than that observed in the DBBM group (134±24 μm). Consistently, the trabecular separation in the SSB group (157±42 μm) was significantly lower (p=0.045) than in the DBBM group (214±33 μm). No significant differences were found in trabecular number of the *de novo* bone between the SSB and DBBM groups (Figure 4c).

**Figure 5:**
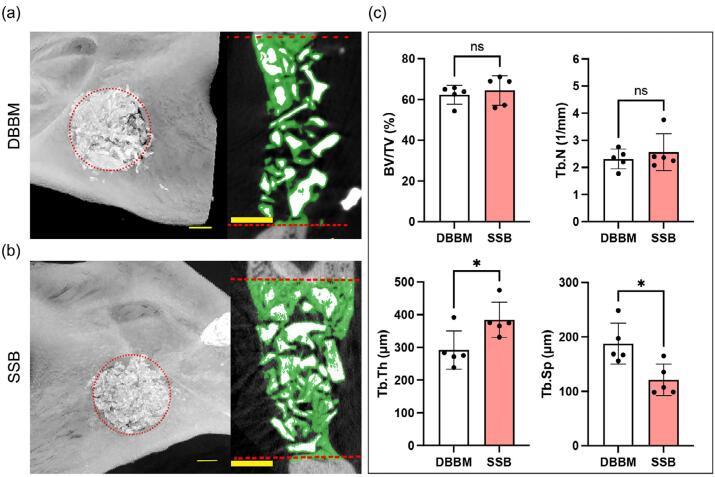
Microcomputed tomography (μCT) of Rat critical-size jawbone defects treated with the SSB, DBBM at postoperative 4 weeks. (a-b) Representative reconstructions from μCT are shown for the rat critical-size jawbone defect treated with DBBM (a) and SSB (b); green color indicates the newly formed bone in the defect area. Scale bar = 1mm; (c) Quantitative analysis of bone volume/tissue volume (BV/TV), trabecular thickness (Tb. Th), trabecular separation (Tb. Sp), and trabecular number (Tb. N) in defect sites treated with DBBM and SSB (n=5). Student’s t test was used for the statistical analysis. ns indicates no significance. *p<0.05.

After four weeks, both blank and gelatin gel group still showed non-union in the defect, although new bone formation was observed at the edges of the defects. In contrast, the SSB and the DBBM groups demonstrated complete filling of the defects (Figure 5a-b). Quantitative analysis revealed that the *de novo* bone volume fraction in the SSB and DBBM groups was 64.4±7.22% and 62.26±4.62%, respectively, with no statistically significant difference between the two (p=0.5920). Consistent with the comparable bone/volume fraction (BV/TV), there were no significant differences in trabecular number or trabecular thickness of *de novo* bone between the SSB and DBBM groups. Notably, the trabecular separation (Tb. Sp) in the SSB group (121±29 μm) was significantly lower (p=0.014) than that in the DBBM group (188±38 μm) (Figure 5c).

**Figure 6:**
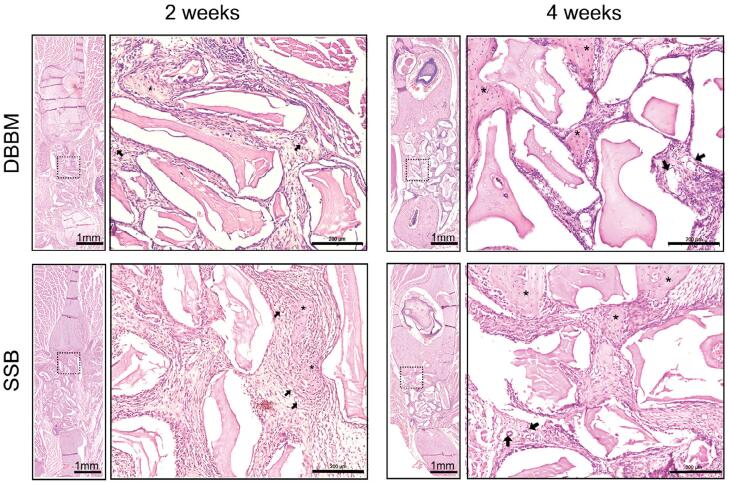
Hematoxylin and Eosin (H&E) Staining of defect samples treated with DBBM or SSB on postoperative 2 weeks and 4 weeks. Black arrows indicated blood vessels. Asterisks indicated newly formed osteoid. Scale bar was 1 mm and 200 μm for low and high magnification, respectively.

**Figure 7:**
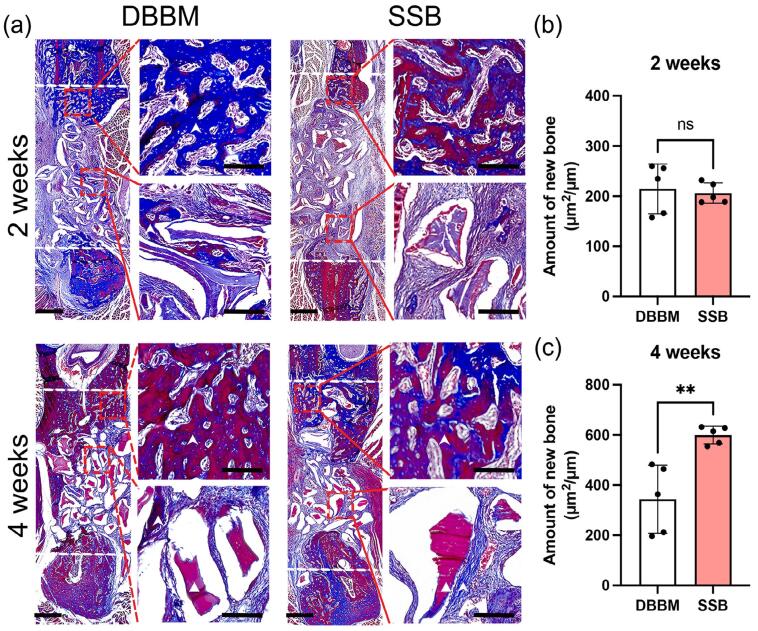
Masson trichrome staining and histomorphometric analysis of defects treated with DBBM and SSB. (a) Representative Masson trichrome staining images of defects with different treatments at postoperative 2 weeks and 4 weeks. White dash line indicates the border of the defect. While triangle indicates the material remnants; White arrow indicates the newly formed bone. Scale bar was 1 mm and 200 μm for low and high magnification, respectively; (b-c) Histomorphometric analysis for the amount of new bone formation using Masson trichrome staining images at postoperative 2 weeks (b) and 4 weeks (c). The amount of bone formation was determined = The bone formation area in the defect area (μm2) / cross-sectional distance (μm). Student’s t test was used for the statistical analysis. ns indicates no significance. **p<0.01.

We further conducted HE and Masson Trichrome staining to evaluate *in vivo* osteogenesis and angiogenesis. No signs of inflammation were observed in any of the four experimental groups (Supplementary Figure 2). After two weeks, samples from both the DBBM and SSB groups showed scattered newly formed osteoid, along with abundant mineral particles in the defect area, accompanied by neovascularization adjacent to the osteoid (Figure 6). Additionally, the blue staining in the Masson trichrome assay evidenced that there was a fibrous callus formation in the center of the defect for both the DBBM and SSB groups. Notably, newly formed bone (indicated by red staining) was particularly observed at the edges of the defect area in the SSB group (Figure 7a). Histomorphometric analysis revealed no significant difference (p=0.7249) in the amount of bone formation between the DBBM and SSB groups (DBBM: 214.8±49.44 μm^2^/μm vs. SSB: 206±20.35 μm^2^/μm) (Figure 7b). At four weeks, more pronounced bone formation with prominent newly formed trabecular structures was observed in the center of the defect area (Figure 7a). Furthermore, histomorphometric assessment showed that the amount of bone formation in the DBBM and SSB groups was 343.0±135.6 μm^2^/μm and 599.8±35.33 μm^2^/μm, respectively (p=0.0035) (Figure 7c).

## Discussion

DBBM is one of the most reliable and effective bone grafting materials in implant dentistry to facilitate successful bone regeneration. However, the currently available DBBM particles show insufficient plasticity and space maintenance, which impedes their application in the repair of extensive bone defects.^6^ There is a substantial need for bone grafting materials that are easy to deploy surgically and that promote a robust osteogenic response.

Recent studies have explored the combination of DBBM particles and PRF, referred to as “sticky bone” to address such clinical challenges. However, concerns regarding technique sensitivity and the lack of standardization in the use of PRF raise questions about its effectiveness in large defects.^8^ Therefore, advancements that enhance the plasticity and stability of DBBM while leveraging its osteoconductivity could significantly improve its clinical application, particularly in irregular and complex bone defects.

Inspired by the sticky bone technique, we created “synthetic sticky bone (SSB)”, which is a binary paste-like composite material composed of DBBM particles and gelatin-based colloidal gel. Microscopic assessment revealed adhesive interaction between gelatin and DBBM particles, which was like previous reports^10^ that demonstrate the existence of adhesive interaction between bisphosphonate-free gelatin and Bioglass^®^. Such adhesive interactions may arise from van de Waals and hydrophobic forces, as well as from local electrostatic attraction between the carboxyl or amine groups of gelation and cations of DBBM particles.^10^

Furthermore, SSB, which is an off-the-shelf and easily prepared binary composite material, demonstrated enhanced injectability and handling properties, making it convenient and effective in defect healing, particularly in treating irregular defects. We further tested the bone regenerative capacity of the SSB in a rat critical-size defect model without using a barrier membrane. This approach enabled us to investigate not only bone regeneration at the end point but also the cohesiveness and stability of various materials throughout the study. When tested *in vivo*, we observed a significantly greater amount of newly formed bones in defects treated with the SSB compared to those treated with DBBM particles or bare gelatin gel. One possible explanation for this finding is that SSB showed better bulk structural integrity than DBBM particles and gelatin gel alone, providing long-term mechanical support that aids new bone formation. Additionally, the gelatin-based colloidal gel created a highly hydrophilic microenvironment owing to their high-water retention capacity,^13^ which favors nutrient exchange and supports osteoprogenitor cells attachment and survival^23-25^ within the tightly packed DBBM particles. Moreover, the gelatin-based colloid gel has been shown to effectively bind osteogenic growth factors,^26^ and facilitate neovascularization,^27,28^ potentially accelerating early osteogenesis.^29-34^ The results suggest that the SSB binary composite material can enhance osteoconductivity, promoting a proper bone-material interface connection and ultimately facilitating bone regeneration.

Given its ease of preparation, paste-like handling properties, and enhanced regenerative performance, the SSB shows strong potential for clinical transformation, particularly in addressing complex irregular bone defects, in which conventional DBBM granules and techniques may encounter challenges. Furthermore, the synthetic nature of the colloidal gel enables standardized and off-the-shelf preparation without autologous blood products or specialized equipment, thus making it more feasible and consistent for regular clinical application.

Note that we used rats as the lowest species because they could provide a critical size of jaw bone defect (diameter =4 mm) with similar biological characteristics that closely resemble human ones.^14^ Despite the significantly higher osteogenic capacity demonstrated by SSB at both two and four weeks, the exact underlying mechanisms of action remain unclear, and the long-term effects on *in vivo* bone formation have yet to be fully elucidated. Furthermore, we only compared the SSB to the DBBM, which is a clinically standard material with well-documented osteoconductive properties. Future clinical evaluations of this composite grafting material, compared to PRF-enriched grafts or autografts, are necessary to further validate its clinical efficacy.

## Conclusions

We successfully developed “synthetic sticky bone (SSB)”, a paste-like composite material composed of DBBM particles and clinical grade absorbable hemostatic gelatin-based colloidal gel. The SSB demonstrated enhanced injectability and plasticity, ensuring the convenience and effectiveness of complete defect filling. When tested *in vivo* in critical-size jawbone defect of rats, the SSB showed superior bulk structural integrity compared to both DBBM particles and bare gelatin gel, providing long-term mechanical support that aids new bone formation. Overall, our results highlighted the importance of material properties such as injectability, plasticity, and mechanical stability in stimulating bone regeneration, which potentially opens up new avenues for bone regeneration therapies.
